# Biomolecular Aspects of Reelin in Neurodegenerative Disorders: An Old Candidate for a New Linkage of the Gut–Brain–Eye Axis

**DOI:** 10.3390/ijms26157352

**Published:** 2025-07-30

**Authors:** Bijorn Omar Balzamino, Filippo Biamonte, Alessandra Micera

**Affiliations:** 1 Research and Development Laboratory for Biochemical, Molecular and Cellular Applications in Ophthalmological Sciences, IRCCS-Fondazione Bietti, 00184 Rome, Italy; bijorn.balzamino@fondazionebietti.it; 2 Fondazione Policlinico A. Gemelli IRCCS-CeMAD Translational Research Laboratories-Digestive Disease Center CeMAD, Department of Medical and Surgical Sciences, 00168 Rome, Italy; filippobimo@libero.it

**Keywords:** Reelin, AD, AMD, neurodegeneration, parainflammation, PNEI, gut–brain axis, eye system, inflammaging, amyloid-β, tau protein

## Abstract

Recent findings highlight that Reelin, a glycoprotein involved in neural development, synaptic plasticity, and neuroinflammation, plays some specific roles in neurodegenerative disorders associated with aging, such as age-related macular degeneration (AMD) and Alzheimer’s disease (AD). Reelin modulates synaptic function and guarantees homeostasis in neuronal-associated organs/tissues (brain and retina). The expression of Reelin is dysregulated in these neurological disorders, showing common pathways depending on chronic neurogenic inflammation and/or dysregulation of the extracellular matrix in which Reelin plays outstanding roles. Recently, the relationship between AMD and AD has gained increasing attention as they share many common risk factors (aging, genetic/epigenetic background, smoking, and malnutrition) and histopathological lesions, supporting certain pathophysiological crosstalk between these two diseases, especially regarding neuroinflammation, oxidative stress, and vascular complications. Outside the nervous system, Reelin is largely produced at the gastrointestinal epithelial level, in close association with innervated regions. The expression of Reelin receptors inside the gut suggests interesting aspects in the field of the gut–brain–eye axis, as dysregulation of the intestinal microbiota has been frequently described in neurodegenerative and behavioral disorders (AD, autism, and anxiety and/or depression), most probably linked to inflammatory, neurogenic mediators, including Reelin. Herein we examined previous and recent findings on Reelin and neurodegenerative disorders, offering findings on Reelin’s potential relation with the gut–brain and gut–brain–eye axes and providing novel attractive hypotheses on the gut–brain–eye link through neuromodulator and microbiota interplay. Neurodegenerative disorders will represent the ground for a future starting point for linking the common neurodegenerative biomarkers (β-amyloid and tau) and the new proteins probably engaged in counteracting neurodegeneration and synaptic loss.

## 1. Introduction

Reelin is a glycoprotein critical for neuronal circuit formation and development, synaptogenesis, and adult synaptic plasticity, playing significant roles in the central nervous system (CNS) and retina in relation to physiological and pathological states [1,2,3]. Low Reelin levels can lead to synaptic dysfunction and neurodegeneration [1]. Alzheimer’s disease (AD) and other dementias represent strong contributors to global disability, as the number of dementia-related disability-adjusted life years (DALYs) rose to over 25 million in 2019 [4]. Age-standardized DALY rates have changed within age-standardized DALY regions, increasing dramatically in high- and middle-Sociodemographic Index (SDI) areas [5]. The 176% DALY increase that occurred between 1990 and 2021 underlines the necessity to improve prevention and care tools for people over 65 such as diagnostics, therapeutics, and policy interventions especially targeting neurodegenerative diseases [6]. 

Emerging evidence links Reelin to AD, a neurodegenerative disease characterized by the deposition of amyloid-beta (amyloid-β or Aβ) plaques and tau tangles in the brain [7]. Several human studies describe altered Reelin levels in AD: notably, the 180 kDa N-terminal Reelin fragment is significantly elevated in the cerebrospinal fluid (CSF) of AD patients compared to those with mild cognitive impairment (MCI) or healthy controls [8]. This raises the possibility that monitoring Reelin and its cleavage products in CSF or blood could help identify individuals progressing from MCI to AD [9].

In the visual system, mice lacking Reelin are impaired in retinal lamination during development [10]. In Reelin-deficient mice, a significant glial activation is detected in the retinas and coupled to signs of neuroinflammation, and particularly reactive Müller cells synthesize and release neurotrophic factors such as NGF and IL33 for neurotrophic compensation and retaining of retinal integrity [11]. Other studies report some altered Reelin levels in humans with AD, pointing at the elevated levels of 180 kDa N-terminal Reelin fragment in the CSF of AD patients compared to those with MCI or healthy controls [8]. This finding would suggest that monitoring Reelin and its cleavage products in CSF or the blood matrix may help in identifying individuals progressing from MCI to AD [9].

One proposed link between age-related macular degeneration (AMD) and AD is abnormal protein accumulation and overt Aβ deposition [12]. Studies have suggested that the amyloid cascade can play a role in the development of AMD, although the hypothesis requires further validation [13]. The formation of senile toxic plaques composed of Aβ aggregates and neurofibrillary tangles formed by tau protein represents a typical hallmark of neurodegeneration [14]. In this context, the intracellular calcium concentration and the ionic strength conditions represent two important drivers of fibril formation and the subsequent onset of progression of neurodegeneration [15,16]. Under high-calcium physiological conditions, S100A9 spontaneously forms fibrils, suggesting a unique aggregation mechanism with potential implications for Alzheimer’s-related neuroinflammation, as detected by atomic force microscopy [15]. Additionally, α-synuclein amyloid fibrils exhibit marked polymorphism depending on protein concentration and ionic strength, highlighting how subtle environmental variables can profoundly influence fibril structures relevant to neurodegenerative pathologies [16]. These pathological accumulations, along with synaptic and neuronal loss, are associated with signs of neuroinflammation, where reactive Müller cells and activated microglia can contribute by releasing a plethora of pro-inflammatory cytokines [17].

At the vascular level, AMD and AD are associated with impaired blood–brain or blood–retina barriers, contributing to vascular dysfunction, tissue hypoxia, and neurodegeneration [18]. Alterations in lipid metabolism, particularly apolipoprotein E (ApoE), are mainly observed, especially regarding the ApoE4 allele, a well-known genetic risk factor for the onset and/or progression of AD and likewise AMD [19].

In recent years, increasing attention has been devoted to Reelin due to its potential role in AD pathogenesis [20]. A reduction in Reelin levels could lead to insufficient modulation of synaptic plasticity, resulting in cognitive dysfunction [8]. The inhibition of Reelin signaling might be one of the causes of the brain’s inability to maintain communication between neurons, an aspect that might also occur in the retina [21].

In this review we performed a critical analysis of the literature of the last 10 years to highlight the main functional aspects of Reelin glycoprotein in the context of the brain–eye axis and brain–gut–eye axis, considering the microbiome pattern that has recently gained multidisciplinary attention in the brain, gut, and eye departments. An extensive literature review of papers published in a range between 2015 and 2025 was performed based on a standard procedure (PubMed; (https://pubmed.ncbi.nlm.nih.gov/; 19 June 2025), Embase; (https://www.embase.com/; 19 June 2025), and Google Scholar; (https://scholar.google.com/; 19 June 2025)). PubMed search terms included “ocular”, “retinopathy”, “maculopathy”, “Alzheimer disease”, “neurodegeneration”, “parainflammation”, “gut-brain-axis”, “gut-brain-eye-axis”, “inflammageing”, “amyloid-β”, “tau protein”, “personalized medicine”, “biological markers”, and “Psycho-Neuro-Endocrine-Immunology circuit”, either alone or in combination with the search term “Reelin” (accessed on 19 June 2025).

## 2. Reelin: Structure, Function, and Homeostasis

Reelin is a 440 kDa secreted glycoprotein (386 kDa with 18 putative N-linked glycosylation sites) expressed from a large genomic section located on human chromosome 7 [22]. This serine protease is encoded by the *RELN* gene (450 kb spanned by 65 exons) as a large secreted extracellular matrix protein within its physiological surroundings [22]. This glycoprotein is composed of a signal peptide, a region like F-spondin, a unique region with an epitope for the CR-50 antibody, and eight tandem repeats [2,23]. Reelin repeats are about 350–390 amino acids long and contain a central epidermal growth factor (EGF) domain flanked by two homologous sub-repeats [24]. Reelin’s receptor-binding fragment and mutational analyses demonstrate that the recognition mechanism is similar to endocytic receptors [25].

Human and murine Reelin shows protein and gene sequence identities of, respectively, 87.2% and 94.2%, making possible studies of its mechanism of action in experimental models [26]. Reelin is chiefly synthesized by neurons in various brain regions, including the cortex and hippocampus, and exerts a key role through binding to the Apolipoprotein E Receptor 2 (ApoER2) and the Very-Low-Density Lipoprotein Receptor (VLDLR), both belonging to the Low-Density Lipoprotein Receptor (LDLR) family [27]. In VLDR-bearing cells, Reelin modulates the synaptic plasticity, dendritic spine density, and neuronal connectivity, driving correct cognitive functions [28]. More precisely, ApoER2/VLDLR–Reelin binding activates intracellular Src-family tyrosine kinases (SFKs), specifically Fyn and Src, phosphorylating the adaptor protein Dab1 at specific tyrosine residues [29]. Reelin–receptor binding activates Disabled-1 (Dab1) signal protein, which in turn activates downstream signaling pathways [30]. Regarding the cell signal downstream following Reelin–ApoE2/VLDLR binding, the Erk1/2 activation leads to increased p90-RSK phosphorylation and induction of immediate-early gene expression, a property connected to many growth factors [31]. Since Erk1/2 activation is not mediated by the canonical signal transduction pathway, it is highly probable that a non-canonical pathway can also be activated by Reelin during brain development [31]. Upon Reelin binding, various biological functions can be observed, all depending on tissue localization and neuronal and non-neuronal cells, involving the phosphorylation of NF-κB, Phosphatidyl-Inositol 3-kinase (PI3K, via downstream effectors Akt and GSK-3β), AKT, and JAK/STAT [21,32]. On the other side, Reelin–ApoER2 binding allows the selective activation of the NF-κB signal which in turn regulates endothelial homeostasis [21]. Physiological Reelin is abundant in the bloodstream, being fundamental for brain, heart, liver, gut, and eye homeostasis, but it might also work as a pro-inflammatory and pro-thrombotic factor, playing critical roles in inflammatory and autoimmune diseases (multiple sclerosis, Alzheimer’s disease, arthritis, atherosclerosis) and the regulation/dysregulation of cancer cells [21,33].

Regarding its activity, Reelin targets also include integrins and Amyloid Precursor Protein (APP), which in turn activates the central Reelin fragment and interacts with the integrin α3β1 to promote neurite outgrowth [34]. Aberrant APP processing has been associated with Reelin reduction in AD brains [35]. At the cellular level, pathological Reelin levels can promote the recruitment of inflammatory cells and drive inflammation by increasing the vasculature as well as the expression of leukocyte–endothelial adhesion proteins on endothelial cells via the activation of nuclear factor kappa-light-chain-enhancer of activated B cells (NF-κB) and the expression of Vascular cell adhesion protein 1 (VCAM-1), Intercellular Adhesion Molecule 1 (ICAM-1), and E-selectin adhesion molecules [21,36,37]. Reelin depletion decreases the recruitment of circulating leukocytes into different tissues due to decreased adhesion properties of the vascular endothelium and decreased leukocyte extravasation, although no direct effect of Reelin has been reported on leukocytes [21]. Reelin expression following injury suggests important roles in regulating stem cell trafficking in neuronal and non-neuronal tissues following injury, like its role in normal organogenesis [38].

Canonical Reelin signaling also appears to be active in the homeostasis of the intestine and colon, suggesting also Reelin’s potential influence in the pathophysiology of some life-threatening chronic and inflammatory syndromes such as Crohn’s Disease [21]. Due to the constant self-renewal gut process, balance between cell proliferation, differentiation, and apoptosis can occur in gut epithelial cells and myofibroblasts (colonocytes, goblet cells, enteroendocrine cells), most probably linked to ApoER2, VLDLR, and Disabled-1 (Dab1) expression and modulation of the local immune response, parainflammation and inflammaging [39].

## 3. Reelin as Biological Marker

The detection or visualization of Reelin protein can be achieved in brain and retina extracts as well as in biological fluids by using research-grade semiquantitative and quantitative techniques (Western blot, immunohistology–cytochemistry, flow-cytometry, ELISA; [40]). Many commercial ELISA kits have been validated for human and mouse detection, mainly for CSF, plasma, and tissue lysate detection [41]. Reelin may prove to be a potential neurodegenerative disease biomarker through minimally invasive sample collection (blood or CSF) coupled with high-sensitivity immunoassays. Until now there has been little evidence for the use of Reelin detection as a prognostic and/or diagnostic biomarker for neurodegenerative disease. By analyzing the biological fluids and tissue biopsies from neuronal and non-neuronal disorders, the potential contribution of Reelin as a biological marker appeared clear [42]. The RELN-APOER2-DAB1 pathway is key in the pathogenesis of AD, where p-Tau disrupts APOER2-DAB1 signaling, providing a worsening of neurodegeneration [42]. Recently, it has been suggested that Reelin path modifications due to biochemical, genetic, and epigenetic influences might be useful to better understand the progression of AD and allow the targeting of Reelin signals with the opportunity to partially counteract the neurodegenerative process [42]. Another peculiarity of Reelin is its direct interaction with some hormones. Notably, Reelin interacts with T3 and T4 thyroid hormones that are essential for proper brain development; their deficiency during pregnancy can induce severe brain damage (cretinism) and neurological deficits, with psychiatric manifestations (schizophrenia and autism). The damages induced by a decrease in Reelin expression in the developing brain are mainly related to the cerebral cortex and hippocampus. The new proteomic platforms proved to be useful to quantify Reelin levels in neuronal and non-neuronal fluids, suggesting the possibility of assessment in ocular fluids collected during routine ophthalmic procedures such as cataract surgery [43].

### 3.1. Reelin Involvement in Neurodegeneration

**Figure 1:**
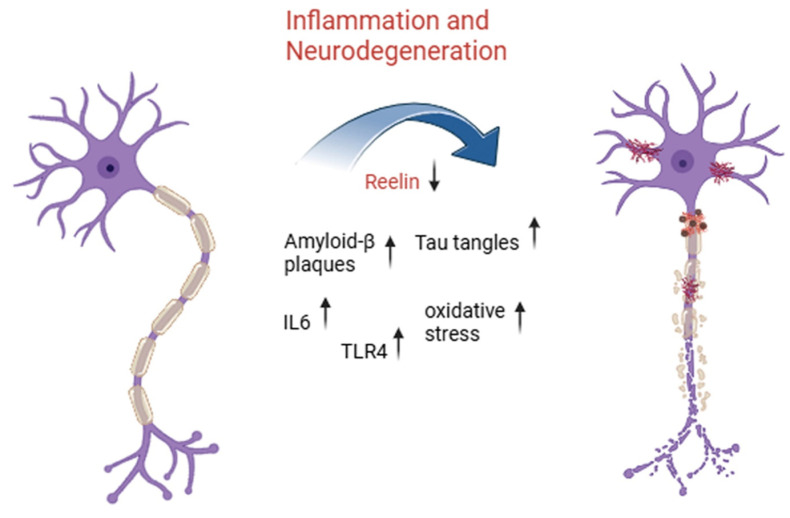
Inflammatory signaling and Reelin downregulation drive neurodegeneration in aging-related disorders. This illustration highlights the progressive transformation of a healthy neuron (**left**) into a degenerating one (**right**) due to chronic inflammatory activation and molecular dysregulation. Central to this process is a reduction in expression of Reelin, a key extracellular matrix protein involved in synaptic plasticity and neuronal integrity. As Reelin levels drop, inflammatory mediators such as interleukin-6 (IL-6) and toll-like receptor 4 (TLR4) become upregulated, promoting a cascade of detrimental events including increased oxidative stress, accumulation of amyloid-β plaques, and formation of neurofibrillary tangles composed of hyperphosphorylated tau protein. Together, these pathological features contribute to the breakdown of neuronal structure and function, ultimately leading to neurodegeneration, a hallmark of aging-related neurodegenerative diseases such as Alzheimer’s disease.

Reelin’s role in AD has sparked interest in terms of whether modulating its activity could help it act as a neuroprotector for damaged neurons [44]. The disruption of Reelin signaling has been recently recognized as a pathological mechanism in AD, as the Reelin–ApoER2/VLDLR pathway regulates tau phosphorylation and amyloid-β metabolism in AD [1,20]. Reduced Reelin levels or impaired Reelin signaling have been associated with severe tau hyperphosphorylation deposition, a route leading to the formation of neurofibrillary tangles [20]. An overview of the role of Reelin and inflammatory markers in the main neurodegenerative process in neurons is shown in Figure 1.

In addition, the protective effects of Reelin were ablated in the presence of ApoE4 (an established genetic risk factor for AD), which modulates the interactions between Reelin and ApoER2 [20]. ApoER2 and VLDLR are undoubtedly important in Reelin’s neuroprotection and may represent therapeutic targets [45]. In ApoE4 carriers, the decreased Reelin signaling associated with impaired mitochondrial functions has been correlated with faster synaptic loss and cognitive decline [22]. On the contrary, an approach to enhancing Reelin signaling or simply boosting Reelin levels has shown potential outcomes of preventing AD progression and counteracting tau pathology by protecting synaptic integrity [22]. The Reelin–ApoER2-DAB1 pathway essentially affects cytoskeletal stability, synaptic plasticity, long-term potentiation, and some immunological signals [46]. Dysfunction of Reelin leads to over-activation of these kinases and hyperphosphorylation of tau protein, hence promoting the assembly of tau into neurofibrillary tangles, and Reelin modulates kinases involved in tau phosphorylation, including glycogen synthase kinase 3 beta (GSK-3β) and cyclin-dependent kinase 5 (CDK5) [47]. Simultaneously, ApoER2 binds to amyloid APP and Aβ precursor, and mutations in the Reelin–ApoER2 pathway can promote amyloidogenic APP processing by overexpression of β-secretase and γ-secretase activity, resulting in higher numbers of amyloid-β peptides [42]. These peptides form aggregates and plaques, which is a characteristic of AD pathology. In addition, the phosphorylation-dependent binding and subsequent degradation of Reelin-modified ApoE isoforms by LRP8 affects the interaction dynamic, and notably, ApoE4 competes with Reelin for this receptor, limiting Reelin signals, leading to increased tauopathy and amyloidogenesis [48].

According to a controversial point of view, Reelin–receptor interaction is a critical neuroprotective mechanism, and impairment of this interaction might contribute directly to the pathophysiological cascade of AD, culminating in Aβ and tau toxic effects [1]. Reduced Reelin levels in CSF might be suggestive of early synaptic dysfunction, while a prompt upregulation of Reelin might delay the neurodegenerative processes in the brain, providing a compensatory response to the neurodegenerative process [42].

### 3.2. Reelin in Anterior and Posterior Eye Segments

Outside the nervous system, Reelin is present in the bloodstream and different organs/tissues, including the eyes, where it plays important roles in the maintenance of visual function [21]. Reelin is physiologically expressed during retinogenesis, but upregulated following ocular injury [49]. At the ocular level, two recent studies have demonstrated that Reelin may rescue some visual deficits, as observed in the retinal layers of a cohort of AD subjects [50].

**Figure 2:**
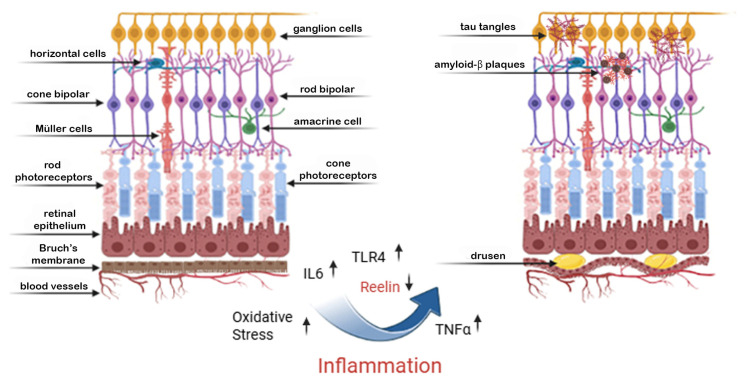
Inflammation-induced retinal degeneration: a shared pathway with neurodegeneration. This figure illustrates the structural and molecular changes in the retina associated with chronic inflammation, paralleling mechanisms seen in cerebral neurodegeneration. On the left, a healthy retina is shown, with organized retinal layers including photoreceptors, Müller cells, bipolar cells, and ganglion cells. On the right, chronic inflammation—marked by elevated IL-6, TNF-α, and TLR4 signaling—leads to increased oxidative stress and downregulation of Reelin, a crucial extracellular matrix protein involved in neuronal stability. These molecular changes promote the accumulation of amyloid-β plaques and tau tangles, not only in the brain but also in retinal neurons, particularly affecting ganglion and bipolar cells. Over time, these pathological processes contribute to the formation of drusen deposits beneath the retinal pigment epithelium and retinal thinning, hallmarks of retinal degenerative conditions such as age-related macular degeneration (AMD). This leads to progressive vision loss in advanced stages, reflecting a shared inflammatory–neurodegenerative axis between the retina and brain.

The retina boasts many similarities to the brain in structure and function. Reelin participates in the differentiation and maturation of retinal neurons, including ganglion cells [51]. Specifically, the protein Reelin has been shown to regulate synapse formation in vision-related brain regions, with altered Reelin levels potentially influencing retinal synaptic plasticity. Reelin plays a critical role in maintaining the structural and functional integrity of the retina [52]. It is expressed in retinal pigment epithelial cells (RPE), photoreceptors, and other retinal layers, where it regulates cell adhesion, extracellular matrix (ECM) dynamics, and synaptic stability [53]. Reelin contributes to the proper alignment of photoreceptors and RPE cells, ensuring efficient phototransduction and waste clearance. Its presence in the retinal microenvironment is also associated with anti-inflammatory properties and protection against oxidative stress, two critical factors in retinal health [40]. People with AD often display retinal dysfunctions, such as impaired reactions to visual stimuli and alterations in retinal ganglions, which might be associated with decreased Reelin [54]. Decreased levels of Reelin in the brain correlate with structural and functional alterations within the retina [21]. As shown in Figure 2, the deregulated expression of Reelin and inflammatory markers may cause AMD in the retina.

Retinal dysfunction can mirror potential pathological events occurring in the brain, including reduced synaptic plasticity or impaired neuronal signaling [3]. Reelin could also modulate neuroinflammation, which plays a pivotal role in AD, and that event could affect the retina, accelerating neuronal damage and visual impairment [8].

Studies suggest that Reelin dysregulation may contribute to AMD development through multiple pathways [8,55]. AMD is characterized by a breakdown of the RPE, accumulation of drusen (lipid-rich deposits), and chronic inflammation in the macula [56]. Decreased Reelin levels may weaken ECM homeostasis, leading to structural damage in the RPE and Bruch’s membrane, abnormal ECM turnover, and accumulation of misfolded proteins in drusen [57]. Reelin changes in AD retinas might provide new perspectives for early diagnosis and tracking disease progression in AD and/or senile dementia [21]. Decreased Reelin levels in experimental retinas have been associated with AD severity [1,58].

As above, the RELN-APOER2-DAB1 complex drives many pathological states [42]. The recent association between ApoE alleles and retinal pathology indicates the importance of recognizing the function of ApoE receptors in normal and diseased retinas [59]. Human Reelin promoter is vulnerable to methylation, and in case of hypermethylation by DNA methyltransferase 1 (Dnmt1), Reelin expression is silenced (negative regulation) [60,61,62]. Reelin can also suppress pro-inflammatory pathways, particularly by downregulating IL-6 and TNF-α expression [8]. In AMD, reduced Reelin activity has been associated with an exacerbated immune response, modulation of cellular responses to oxidative damage, and impaired ability of the retina to manage oxidative stress and lipid metabolism, all aspects contributing to drusen formation and photoreceptor loss (RPE damage) [8,56,63].

Recently, the ocular fluids have been proposed as a good matrix for diagnosis of neurodegenerative disorders in a minimally invasive alternative to CSF, considering their more accessible and reliable diagnostic route in both sampling and analysis of biomarkers [64]. In particular, analysis of aqueous and vitreous humors as well as tears, in connection with direct imaging of the retina, could be a promising, non-invasive or minimally invasive approach to initiate the use of Reelin as an early biomarker for AMD or AD [40,65]. Changes in tear Reelin levels could reflect systemic or brain alterations, providing the link between nervous and visual systems [66]. Since ocular fluids are influenced by the local microenvironment and represent a reservoir of tissue-mediator release, the detection of Reelin levels in aqueous and vitreous humors could correlate with alterations in CSF from AD tissues, offering an alternative method for early biomarker detection [67,68]. This concept is supported by the fact that the retina is part of the CNS and shares common neuropathological features with the neurodegenerating brain [21,69].

Recent studies have been conducted to investigate the interactions of Reelin with other significant molecules, like integrins and matrix metalloproteinases, to find drug targets [33]. Reelin’s role in the maintenance of synaptic stability, its interaction with AMD risk factors, and its presence in biological fluids make it an attractive candidate as a potential early biomarker [21,40]. These methods have the potential to improve diagnostic accuracy, facilitate timely interventions, and offer continuous disease tracking.

### 3.3. Reelin and GUT Axis

The “gut axis” refers to the communication between the gut and other organs occurring in a bidirectional mode and with the interplay of various actors (nerves, portal vein, intestinal epithelial barrier) with the intervention of the gut microbiota [70]. High expression of Reelin–receptor pathways (ApoER2, VLDLr, and Dab1 protein) have been found in both experimental gut models and human diseases [37]. Reelin transcript expression was mainly observed in fibroblasts, while transcripts specific for Dab1, ApoER2, VLDLr, and integrins α3 and β1 were found in enterocytes, crypts, and enteric fibroblasts [57]. Reelin has been isolated from intestinal fibroblasts and the villus epithelial layer (α-smooth muscle actin (α-SMA) expressing myofibroblasts) [71]. The presence of VLDLR and Dab1 proteins was confirmed in both crypt and villus cells, with notable abundance in enterocytes, while ApoER2 protein was identified in the upper half of villi and absent in crypt structures [57] within the enteric nervous system due to analogous processes of proliferation, differentiation, and apoptosis along the crypt–villus axis [57]. Although the precise mechanisms governing crypt–villus cellular migration have not been fully elucidated, they are postulated to incorporate a synergistic interplay of cell–cell adhesion, cell–matrix adhesion, and alterations in the cytoskeleton. Empirical research has provided evidence suggesting that Reelin may also influence cellular migration along the crypt–villus axis, being higher in the crypt area compared to the villi [57]. This variation can potentially be explained by the release of Reelin at the basement membrane, followed by its diffusion to the epithelial layer to execute its functions [57]. Collectively, these investigations offer a persuasive argument for Reelin’s involvement in crypt–villus migration. Reeler mice display elevated inflammatory scores compared to wild-type mice, suggesting that the mutation heightens their vulnerability to DSS-induced colitis [72].

**Figure 3:**
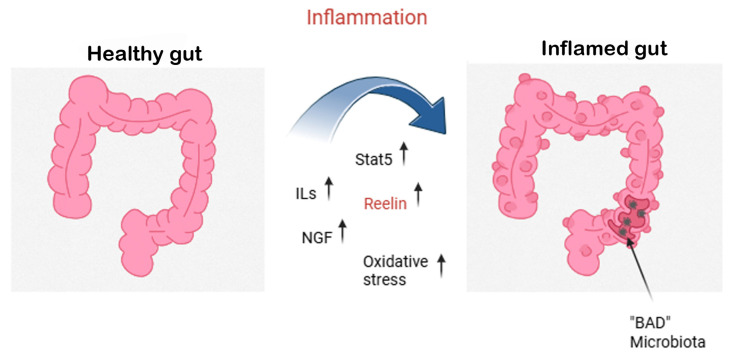
Gut inflammation and the neuroprotective role of Reelin and NGF. This figure explains the shift from a healthy gut (**left**) to an inflamed gut (**right**) driven by dysbiosis and excessive inflammatory responses. During gut inflammation, pro-inflammatory cytokines (interleukins, ILs) and oxidative stress levels rise, leading to epithelial damage and a loss of beneficial (“good”) microbiota. In turn, this allows colonization by pathogenic (“bad”) microbiota, perpetuating the inflammatory loop. Molecular mediators such as Signal Transducer and Activator of Transcription 5 (STAT5) are activated, while the expression of Reelin, a glycoprotein with known neuroprotective functions, is upregulated. Simultaneously, nerve growth factor (NGF) and residual Reelin act as compensatory signals, helping to mitigate inflammation and protect the gut’s neuronal and epithelial integrity.

In recent work by Carvajal and coauthors, evidence that the mouse distal colon upregulates Reelin production in response to dextran sodium sulfate (DSS)-colitis through DNA Methyltransferase 1 (DNMT1)-dependent hypomethylation of the gene promoter region and that Reelin offers protective effects against colitis has been reported [73]. The gut microbiota, a complex ecosystem that influences digestion, immune response, and metabolism, has been linked to health and well-being, including the absence of degenerative diseases [74], while inflammation can cause gut pathologies, as shown in Figure 3.

These mechanisms mirror those observed in the retina and brain, where Reelin downregulation, inflammation, and oxidative stress contribute to neurodegenerative changes. The presence of common molecular actors in these organs supports the existence of a brain–eye–gut axis, where neuroinflammatory and neuroprotective signals are interlinked across systems. Understanding this axis may offer novel targets for treating chronic diseases that span multiple organs, including neurodegenerative, retinal, and gastrointestinal disorders.

## 4. Reelin in the Psycho–Neuro–Endocrine–Immunology Circuit and Gut–Brain–Eye Axis

The Psycho–Neuro–Endocrine–Immunology (PNEI) discipline, also known as Psycho–Neuro–Immunology or PNI, studies the interactions between the nervous, endocrine, and immune systems, and the mechanisms of action involved in modulating PNEI balance in humans [75]. Studies on the possible link between these four systems started in the first decades of the last century with the pioneering work of several scientists who initiated the interest in the chemical communication between the brain and the endocrine glands. By combining different multidisciplinary findings, PNEI aims to demonstrate that human systems do not work alone, but in a tidy network, leading us to consider that the psyche is a dimension emerging from the biological dimension, with influences on the endocrine, immunological, and visual systems, and possesses its own autonomy that allows it to act back on the brain by modifying it when seemingly non-correlated changes occur in the endocrine or immunological system [76].

To date, PNEI is an innovative interdisciplinary approach with notable influence on precision medicine that might represent a paradigm shift away from a strictly biomedical view of health and disease, towards an interdisciplinary point of view. In the last decade, our group extended its own interest to the PNEI and visual system (PNEI-V) axis, mainly based on NGF and cortisol findings [77,78]. For in-depth study of this topic, we hypothesized a crucial role for PNEI-V in vitreoretinal diseases, hypothesizing some network for restoration. Mediators with pleiotropic effects on neuronal function and cognitive processes such as Reelin, NGF, BDNF, choline, microbiota supplies, and others, as well as combined citicoline, memantine, and acetylcholinesterase inhibitors (AChEIs) in AD patients, suggest the beneficial effects of this way of thinking [79].

## 5. Preclinical Studies and Their Potential Therapeutic Implications

While no therapies directly targeting Reelin have yet entered clinical use, several promising strategies have emerged from preclinical research [37]. The monoclonal antibody CR50 has demonstrated efficacy in treating mice with autoimmune and inflammatory conditions. When applied to experimental autoimmune encephalomyelitis, CR50 selectively abolishes Reelin, regulates endothelial activation, and avoids leukocyte infiltration without affecting synaptic plasticity or toxicity to the whole CNS [37]. As a result of CR50 disrupting Reelin oligomerization, Dab1 cannot interact with Reelin and downstream signaling is not activated in endothelial cells [27]. On the contrary, increasing Reelin has shown promise as a treatment in Alzheimer’s mouse models. This can be achieved either by protecting neurons from tau hyperphosphorylation and Aβ42-induced toxicity in vitro or by overexpressing Reelin in APP-overexpressing animals, which delays the formation of Aβ plaque and maintains memory [1]. Furthermore, Reelin inhibits α-synuclein aggregation and promotes dopaminergic neuron survival in Parkinson’s models by upregulating lysosomal LAMP1, particularly in environments of enrichment. In vitro, CR-50 reverses these effects [80].

Overall, anti-Reelin and Reelin-enhancing therapies show distinct mechanistic efficacy in a variety of neurodegenerative models, with new clinical safety profiles in vivo [37]. Translation to human disease is still difficult, though. Future studies should concentrate on (i) assuring long-term CNS safety, (ii) optimizing dosage and delivery for systemic Reelin modulation, and (iii) starting early-phase human trials to assess biomarkers of endothelial and neuronal protection. Reelin is increasingly recognized for its involvement in neurodegenerative diseases, particularly in AD [21]. Its independent diagnostic utility is, however, limited by inconsistent results from various studies and a lack of specificity. Because of post-translational changes and peripheral expression, blood levels of Reelin seem less informative [27]. Strategies to modulate Reelin therapeutically show that using monoclonal antibodies to deplete Reelin peripherally can reduce neuroinflammation without affecting the function of the central nervous system [37], while improving Reelin signaling has demonstrated neuroprotective effects in AD models and may reduce tau pathology, as demonstrated by a protective *RELN* variant in a human AD-resilient case [22]. However, the available evidence is mainly preclinical and there are no standardized clinical assays for Reelin. Longitudinal human studies, standardization of measurement protocols, and combination of Reelin with established biomarkers are needed for confirming its diagnostic and therapeutic application.

## 6. Conclusions and Future Perspectives

The retina is increasingly recognized as a mirror of brain neurodegeneration, with shared embryologic origin and molecular characteristics [81]. AD and AMD also share many similarities in pathology, such as amyloid-β and tau accumulation, inflammation, and vascular changes. These similarities imply that retinal alterations in AMD may mirror, or even precede, the existence of the same in the brain [82]. However, whether AMD is an early indicator of AD (or vice versa) remains unclear, and further longitudinal studies are needed to straighten out this relationship. Reelin occupies a central role in both neural development and degenerative processes. This large extracellular matrix protein is essential for neuronal migration and synaptic plasticity and survival, and its dysregulation is well documented in AD (co-localizing with Aβ plaques and tau tangles) [57].

In this era characterized by the debate on Xamamine, Citicoline, and microbiota in terms of their beneficial application in AD and DS neuronal loss, we stress the potential use of Reelin first as a biomarker for monitoring early and ongoing inflammatory processes (neuronal loss) [8].

Therapeutically, modulating Reelin signaling is being explored as a novel anti-inflammatory strategy. This suggests a way to reduce chronic neuroinflammation in diseases like AD, multiple sclerosis, atherosclerosis, or arthritis by targeting Reelin’s vascular actions [21]. Alterations in Reelin signaling related to tau hyperphosphorylation and Aβ deposition might reflect Reelin being a surrogate marker useful for detecting conditions from MCI to AD (differential diagnosis). How systemic factors control Reelin expression, secretion, and proteolysis remains largely unknown. Elucidating these regulatory mechanisms is important because Reelin’s effects outside the brain—and its interplay with risk factors like hypertension, hyperlipidemia, or gut microbiome alterations—may profoundly influence neurodegenerative processes [50].

Prospective studies should measure Reelin in blood and CSF alongside established biomarkers (Aβ, tau) to assess its sensitivity, specificity, and added value. It will also be important to control for peripheral variables (age, sex, diet, inflammation) since Reelin circulates in plasma with little variation once adulthood is reached.

Understanding how enzymes, for example a disintegrin and metalloproteinase with thrombospondin motifs (ADAMTS) or metalloproteinases (MMPs), and signaling pathways alter Reelin processing in AD, AMD, and diabetes could reveal new drug targets. Long-term safety profiles of peripheral Reelin depletion must be established to avoid unintended vascular or coagulation side effects. Meanwhile, the potential cognitive benefits of enhancing Reelin signaling in the brain deserve exploration, given experimental evidence that Reelin supplementation can restore synaptic function in AD models.

In summary, Reelin binds retinal and cerebral physiology, and its perturbation is increasingly implicated in AMD, AD, and other chronic inflammatory diseases [21]. Fully decoding Reelin’s multifaceted roles, from development and plasticity to immune modulation, could lead to novel strategies for early diagnosis and intervention. Continued interdisciplinary research combining ophthalmology, neurology, and immunology will be essential to realize the promise of Reelin-based diagnostics and therapeutics in neurodegenerative disease. This will involve clarifying whether retinal Reelin alterations precede or mirror brain pathology in AD/AMD, and validating circulating Reelin (and fragment) assays for early detection of cognitive decline. Therefore, elucidating Reelin’s regulation, cleavage, and function in peripheral tissues (liver, endothelium, intestine) and its impact on neurodegeneration and developing anti-Reelin assay (or Reelin-enhancing) therapies could ensure inflammation modulation without compromising essential CNS Reelin signaling.

Addressing these questions will deepen our understanding of Reelin biology and may lead to novel biomarkers and treatments for Alzheimer’s disease, AMD, and related disorders.

## Data Availability

No new data were created.
